# Anti-PD1 therapy induces lymphocyte-derived exosomal miRNA-4315 release inhibiting Bim-mediated apoptosis of tumor cells

**DOI:** 10.1038/s41419-020-03224-z

**Published:** 2020-12-11

**Authors:** Nina Guyon, Delphine Garnier, Joséphine Briand, Arulraj Nadaradjane, Gwenola Bougras-Cartron, Judith Raimbourg, Mario Campone, Dominique Heymann, François M. Vallette, Jean-Sébastien Frenel, Pierre-François Cartron

**Affiliations:** 1 CRCINA, INSERM, Université de Nantes, Nantes, France; 2 Equipe Apoptose et Progression Tumorale, LaBCT, Institut de Cancérologie de l’Ouest, Saint Herblain, France; 3 Cancéropole Grand-Ouest, Réseau Niches et Epigénétique des Tumeurs (NET), Saint Herblain, France; 4 EpiSAVMEN Network (Région Pays de la Loire), Saint Herblain, France; 5 Department of Medical Oncology, Institut de Cancérologie de l’Ouest site René Gauducheau, Saint Herblain, France; 6 LabEX IGO, Université de Nantes, Nantes, France

**Keywords:** Cancer, miRNAs

## Abstract

Anti-PD1 immunotherapy, as a single agent or in combination with standard chemotherapies, has significantly improved the outcome of many patients with cancers. However, resistance to anti-PD1 antibodies often decreases the long-term therapeutic benefits. Despite this observation in clinical practice, the molecular mechanisms associated with resistance to anti-PD1 antibody therapy have not yet been elucidated. To identify the mechanisms of resistance associated with anti-PD1 antibody therapy, we developed cellular models including purified T cells and different cancer cell lines from glioblastoma, lung adenocarcinoma, breast cancer and ovarian carcinoma. A murine model of lung cancer was also used. Longitudinal blood samples of patients treated with anti-PD1 therapy were also used to perform a proof-of-concept study of our findings. We found that anti-PD1 exposure of T-cell promotes an enrichment of exosomal miRNA-4315. We also noted that exosomal miRNA-4315 induced a phenomenon of apopto-resistance to conventional chemotherapies in cancer cells receiving exosomal miRNA-4315. At molecular level, we discern that the apopto-resistance phenomenon was associated with the miRNA-4315-mediated downregulation of Bim, a proapoptotic protein. In cellular and mice models, we observed that the BH3 mimetic agent ABT263 circumvented this resistance. A longitudinal study using patient blood showed that miRNA-4315 and cytochrome c can be used to define the time period during which the addition of ABT263 therapy may effectively increase cancer cell death and bypass anti-PD1 resistance.This work provides a blood biomarker (exosomal miRNA-4315) for patient stratification developing a phenomenon of resistance to anti-PD1 antibody therapy and also identifies a therapeutic alternative (the use of a BH3 mimetic drug) to limit this resistance phenomenon.

## Introduction

Immune checkpoint inhibitors, in first line or in combination with conventional chemotherapy, have shown great promise as anticancer treatment^1^. Anti-PD1 therapy is, to date, one of the most effective anticancer immunotherapies. Despite this success, a significant number of patients develop, or will develop, resistance to this therapy^2–5^. Innate resistance to anti-PD1 therapy is found in 60% of melanoma patients^6^, and 25% develop resistance after an initial phase of objective response^7^. In non-small-cell lung carcinoma, Gettinger et al. identified patients characterized by a phenomenon of acquired resistance to anti-PD1 therapy^8^. Whereas resistance to anti-PD1 therapy is observed in clinical practice, its molecular causes have not been fully documented. Consequently, extensive researches need to be performed in order to complete the description of biomarkers associated with the resistance to anti-PD-1 therapy. In addition, the description of these innovative biomarkers could provide therapeutic targets against the anti-PD1-induced resistance.

Due to the fact that anti-PD1 therapy targets lymphocytes and the efficiency of anticancer therapy is measured by the impact on the tumor cells, we postulated that studying the molecular mechanisms of resistance of anti-PD1 therapy should take into consideration existing intercellular communication between lymphocytes and tumor cells. As exosomes are the carriers for the intercellular transfer of the miRNA responsible of chemoresistance^9–12^, we herein investigated whether exposure of T cells to anti-PD1 therapy might promote the expression of exosomal miRNA (exomiR) causing the chemoresistance of cancer cells.

## RESULTS

### The exosomes of T cells exposed to anti-PD1 therapy decreased temozolomide-induced cell death via miR-4315

**Fig. 1:**
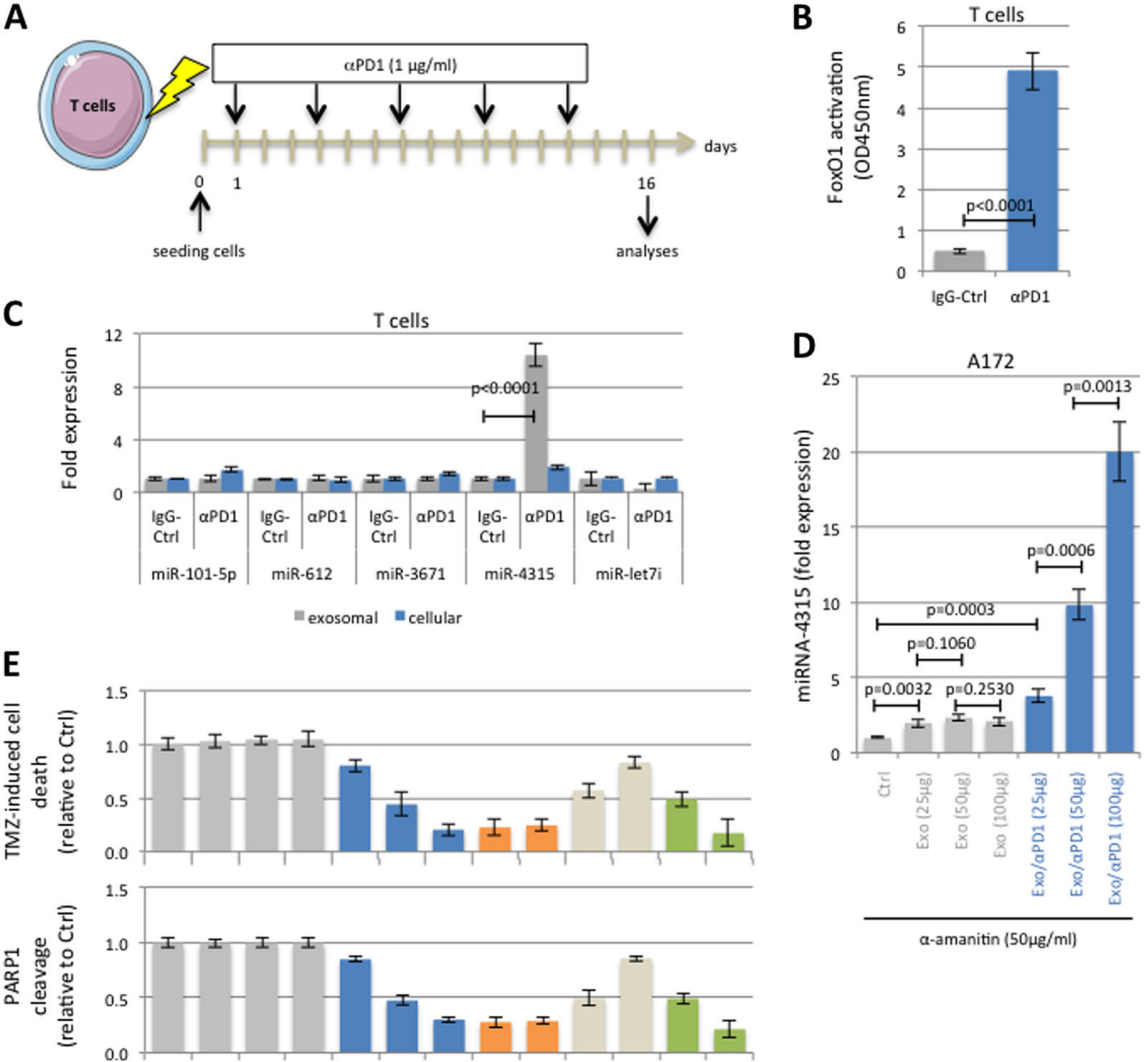
Exosomes of T cells exposed to anti-PD1 therapy decrease the temozolomide-induced cell death via miR-4315. **A** Schematic representation of T cell exposure to anti-PD1 (αPD1, Pembrolizumab, Biovision, France). **B** On day#14, TransAM® FKHR/FOXO1 kit (Active Motif, France) was applied to note that anti-PD-1 exposure activates FoxO1. **C** On day#14, RT-qPCRs were performed to show that anti-PD-1 exposure increases the exosomal level of miR-4315. **D** RT-qPCRs were performed to validate the miR-4315 levels in A172 cells after their exposure to indicated exosomes. **E** Impact of exosomal pre-treatment on the TMZ-induced apoptosis level.

The effect of anti-PD1 antibody (αPD1) therapy on T cells was analyzed by exposing purified human T cells to αPD1 (Fig. 1A). It has been previously demonstrated that anti-PD1 therapy promoted the transcriptional activity of FoxO1 in T lymphocytes^1^. In our model, the transcriptional activity of FoxO1 in T cells treated with 1 μg/ml of αPD1 strongly increased (*p* < 0.0001) (Fig. 1B). We thus analyzed the expression of five FoxO1-regulated miRNA (miR-101-5p, miR-612, miR-3671, miR-4315, miR-let7i) according to the predictive study performed with the miRGen.v3 program. RT-qPCR confirmed the expression of these five miRNA in T cells (Fig. 1C) and αPD1 treatment did not appear to modify their expression. However, strikingly, miR-4315 was ten times more expressed in exosomes derived from T cells exposed to αPD1 (Exo/αPD1) than in exosomes derived from T cells exposed to the IgG control (Exo) (Fig. 1C).

We then analyzed the effect of the exosomes produced by the control or αPD1-treated T cells on the glioma cell line A172. RT-qPCR indicated that the intracellular level of miR-4315 in A172 glioblastoma cells increased in a dose-dependent manner in the presence of Exo/αPD1, α-amanitin and the exosomes derived from T cells exposed to the IgG control (Exo) (Fig. 1D). This observation suggested a possible uptake and transfer of miR-4315 from the Exo/αPD1 to the tumor cells (as α-amanitin was used to block the de novo production of miR-4315).

Temozolomide (TMZ) is the standard chemotherapeutic agent used to kill the glioblastoma (GBM) cells. We therefore studied the TMZ effect on A172 in the presence or absence of Exo/αPD1 by measuring its cytotoxic effect and its ability to induce apoptosis through the detection of the cleaved forms of PARP1 and Caspase-3 (that are two apoptosis biomarkers). In this experiments, cells were exposed for 48 h to indicated exosome previous to be treated with TMZ (50 μM, 72 h). Thus, we noted that Exo/αPD1 limited the TMZ-induced apoptosis (Fig. 1E). To determine the contribution of miR-4315 in this process, Exo/αPD1 was transfected with an anti-miR-4315. Fig. 1E shows that the presence of anti-miR-4315 significantly reduced the TMZ-resistance associated with the addition of Exo/αPD1. Overall, our data demonstrated that exosomal miR-4315 limits the apoptosis induced by a chemotherapeutic drug. In addition, this effect is similar to that one seen with miR-4315 alone (Fig. 1E).

### Exposure to anti-PD1 promotes a phenotype of chemotherapy resistance in several cancer cell types via the exomiR-4315/Bim axis

**Fig. 2:**
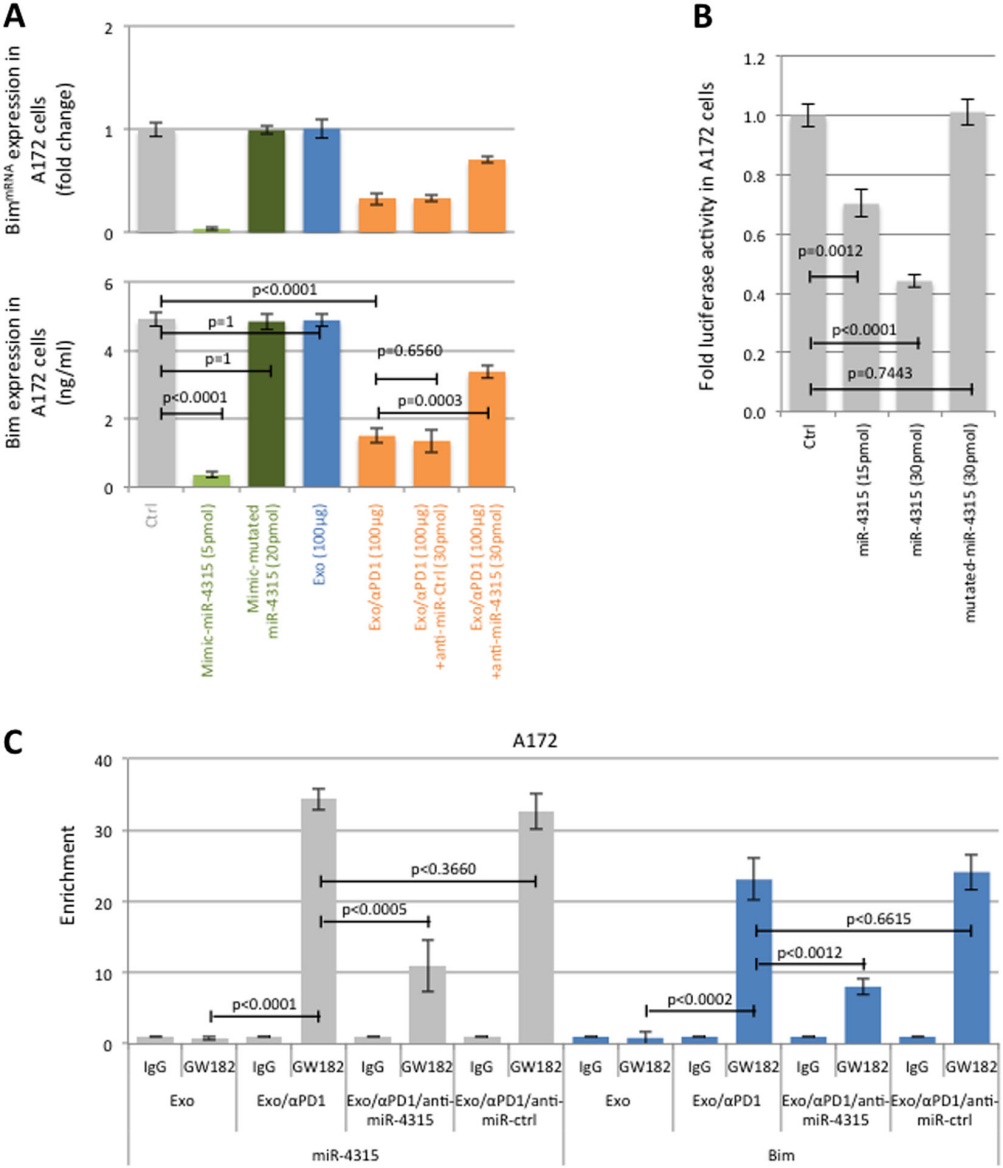
Exosomal miR-4315 targets Bim. **A** RT-qPCR and ELIZA were used to analyze the impact of mimic miR-4315, exosomes derived from T cells exposed to the IgG control (Exo) and exosomes derived from T cells exposed to αPD1 (Exo/αPD1) ± antimiR. **B** The luciferase reporter assay was used to verify that Bim was the target gene of miR-4315. **C** The graph illustrates the miR-4315 and 3′UTR/Bim enrichments on GW182 and IgG (negative control). Experiments were performed using the RiboCluster Profiler kit (CliniScience, France) according to the manufacturer’s instructions. Experiments were performed 48 h after exosomal exposure.

Giving our previous data indicating that exosomal miR-4315 limits apoptosis, we postulated that this miR could target a proapoptotic protein in the BCL2 family as these proteins are central to the execution of the apoptosis. The Target Scan Human website suggests that Bim, a proapoptotic protein, could be a miR-4315 target (Figure S1). We thus focused our study on this protein. A mimic miR-4315, though not an inactive mutant, downregulated Bim at protein and mRNA levels (Fig. 2A) and decreased the luciferase activity associated with the 3′UTR/Bim plasmid in A172 (Fig. 2B). Exo/αPD1 decreased Bim expression in A172 cells and anti-miR-4315 significantly limited the decrease in Bim expression induced by Exo/αPD1. GW182-CLIP-qPCR indicated that miR-4315 were co-immunoprecipitated with 3′UTR/Bim in A172 cells treated with T-cell-derived Exo/αPD1, but not with T-cell-derived Exo (Fig. 2C). Likewise, anti-miR-4315 also decreased the level of 3′UTR/Bim and miR-4315 co-immunoprecipitated with GW182, while an anti-miR-Ctrl had no effect (Fig. 2C).

**Fig. 3:**
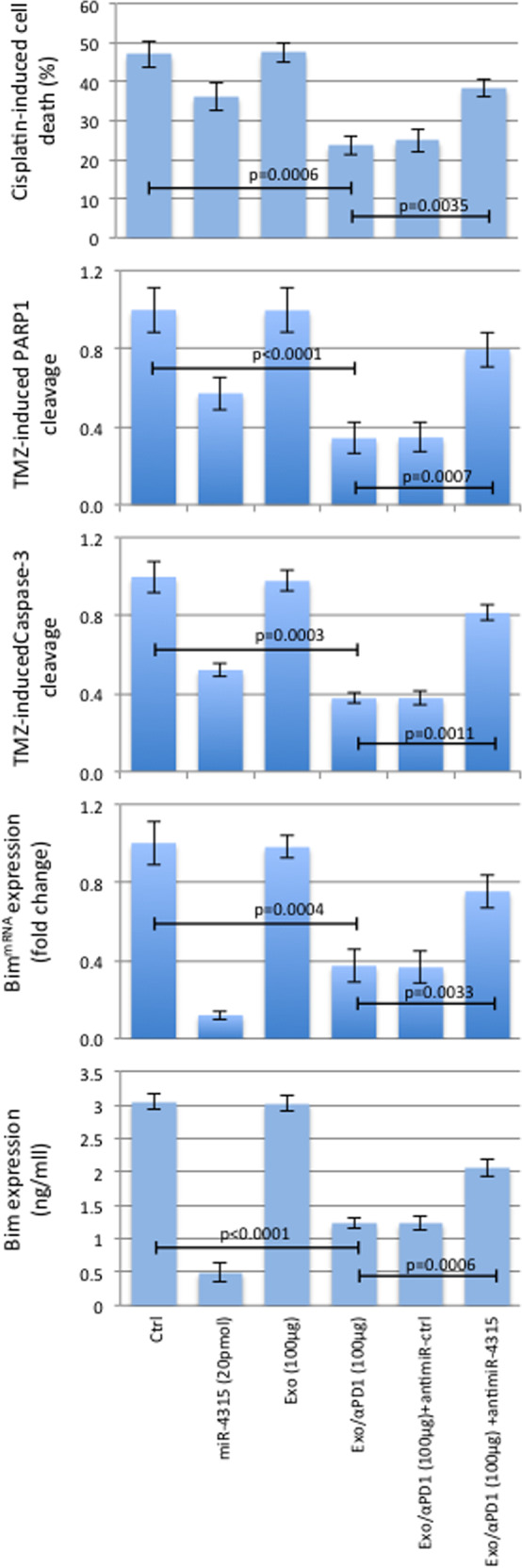
Exosomes derived from T cells exposed to αPD1 (Exo/αPD1) promote a phenotype of cisplatin-induced apoptosis in A549 cells via miR-4315. Cisplatin-induced cell death measure, PARP and Caspase-3 cleavages were applied to show that exosomes derived from T cells exposed to αPD1 (Exo/αPD1) promote a phenotype of cisplatin-induced apoptosis. RT-qPCR and in-cell ELIZA were applied to show that this phenomenon is associated with the miR-4315-mediated down-regulation of Bim.

Similar investigations have been carried out on A549 (a lung cancer cell line), OV90 (an ovarian cancer cell line), and MCF7 (a breast carcinoma cancer cell line) treated or not with oxaliplatin, cisplatin and paclitaxel, the respective chemotherapy for each of these cancers. As found with the A172 glioma cell line, Exo/αPD1 decreased the cell death induced by each drug and these effects were counteracted by an anti-miR-4315 (Fig. 3 and Supplementary Fig. S2). As expected, cell death inhibition was associated with downregulation of the Bim expression and the decrease of PARP and Caspase-3 cleavage (Fig. 3 and Supplementary Fig. S2).

Here, we demonstrated that exosomes derived from T cells exposed to αPD1 decreased Bim expression through exomiR-4315. The incorporation of miR-4315 into cancer cell lines in turn led to increased resistance to chemotherapy through the down regulation of apoptosis.

### The longitudinal expression of exomiR-4315 is associated with a serum biomarker of apopto-resistance in lung cancer patients treated with anti-PD1 therapy

**Fig. 4:**
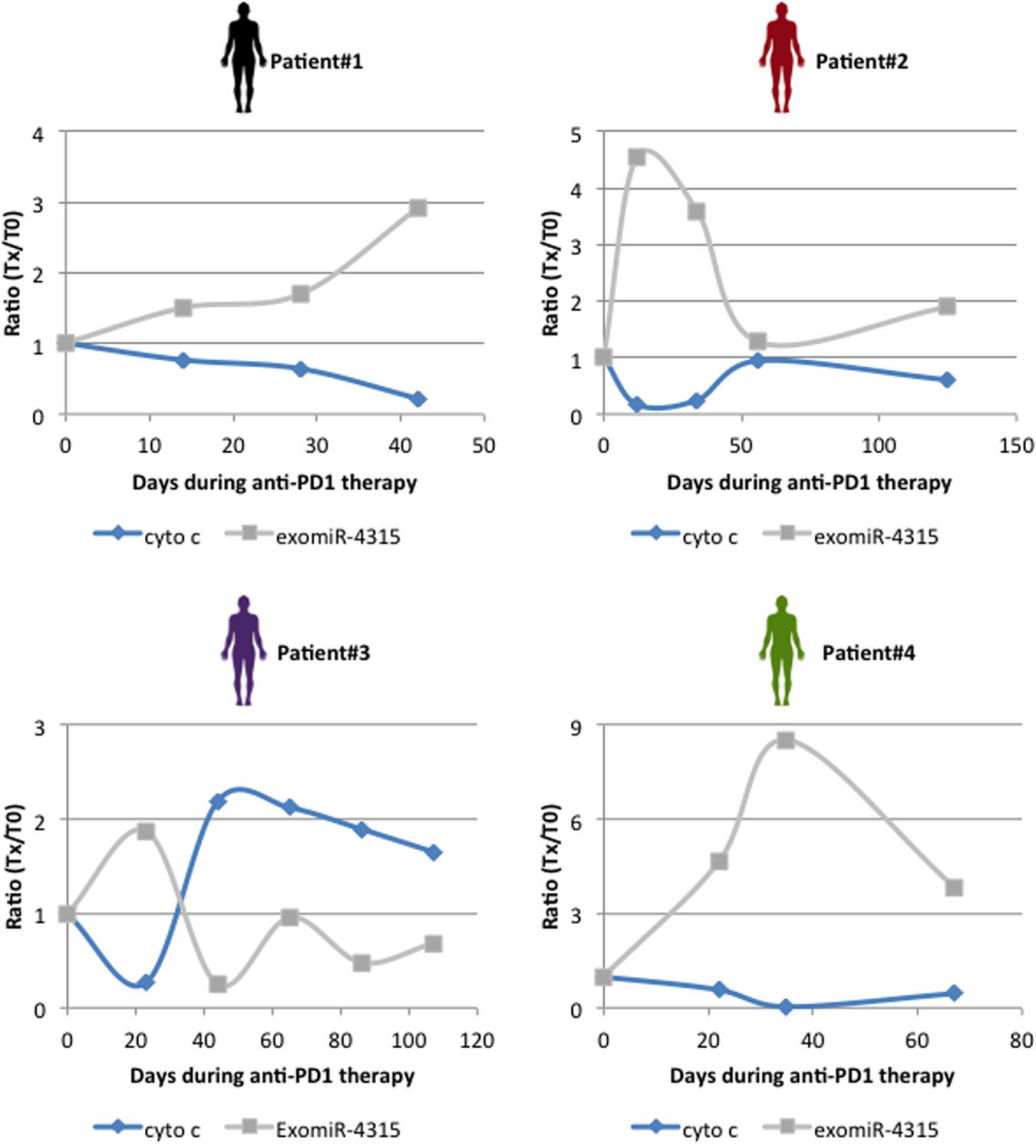
The longitudinal expression of exomiR-4315 is associated with a serum biomarker of apopto-resistance in lung cancer patients treated with anti-PD1 therapy. Graphs illustrate the longitudinal evolution of exomiR-4315 expression and blood cytochrome c in four lung cancer patients treated with anti-PD1 therapy (one patient per graph) (Expressed as a ratio at measurement day compared to T0).

To determine the clinical relevance of our observations, we examined the exomiR-4315 expression and serum cytochrome c concentrations in four patients with lung cancer treated with αPD1 (Supplementary Fig. S3). Serum cytochrome c concentration was chosen as a cell death biomarker^13^. The serum levels of exomiR-4315 and cytochrome c were dynamically regulated throughout the anti-PD1 treatment (Fig. 4). By comparing the exomiR-4315 expression between two administrations of αPD1 in patients, we noted that the exomiR-4315 expression increased in 10/15 cases. We also observed that exomiR-4315 and serum cytochrome c levels had antiparallel or mirrored evolution throughout the administration of αPD1 in patients (Fig. 4). Furthermore, Pearson’s correlation test revealed that longitudinal exomiR-4315 expression was inversely correlated with serum cytochrome c concentrations in all patients recruited (Fig. 4 and Supplementary Fig. S4). Overall, these data demonstrate that the longitudinal expression of exomiR-4315 was inversely correlated with a serum biomarker for apoptosis resistance in lung cancer patients treated with αPD1.

### ABT263 abrogates the anti-PD1/exomiR-4315-induced resistance to chemotherapy in an in vivo model of lung cancer

**Fig. 5:**
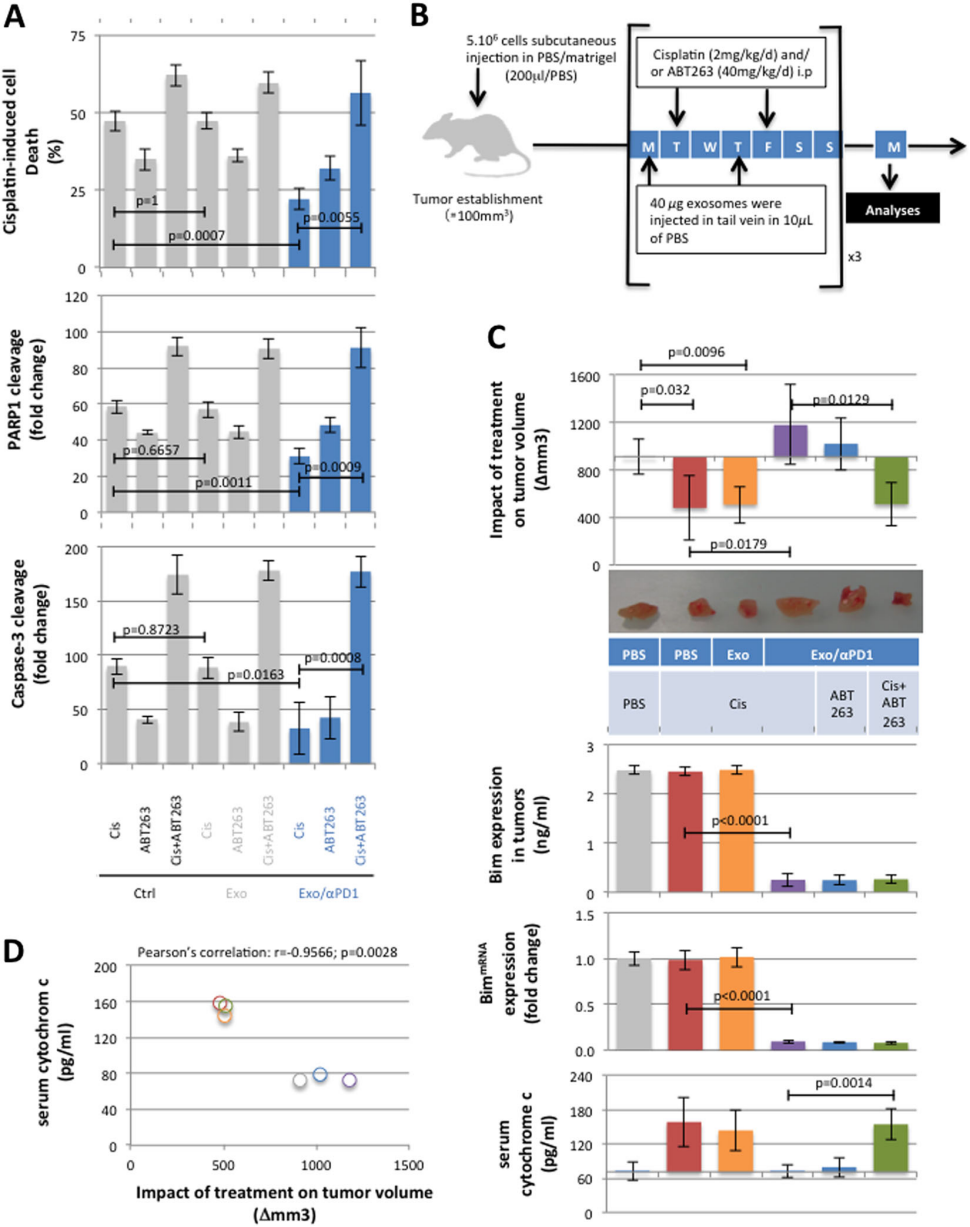
ABT263 abrogates anti-PD1/exomiR-4315-induced resistance to chemotherapy in an in vivo model of lung cancer. **A** Cisplatin-induced cell death measure and PARP and Caspase-3 cleavage studies were applied to show that the phenotype of cisplatin resistance induced by exosomes derived from T cells exposed to αPD1 (Exo/αPD1) was abrogated by the use of ABT263.A Cell. **B** Schematic representation of our in vivo experimentations. **C** Graph represents the impact of treatment on tumor volume, Bim expression at mRNA (RT-qPCR experiments) and protein (ELIZA, Bim ELIZA Kit MyBioSOURCE#MBS9500064, USA) levels and on the level of serum cytochrome c (Cytochrom C ELIZA kit, Biovision#E4286-100, France). Each treatment included four mice. **D** Correlation between the impact of treatment on tumor volume and the level of serum cytochrome c in mice.

The dynamic and antiparallel expression of the serum levels of exomiR-4315 and cytochrome c suggests that the effectiveness of a treatment in inducing cancer cell death can fluctuate through different phases of effectiveness and ineffectiveness to promote cell death throughout the therapy. In view of the data described above, associating the ineffectiveness of cisplatin + αPD1 therapy with exomiR-4315-induced Bim down-expression, we then studied the effect of a “Bim/BH3 mimetic drug” such as ABT263 on the resistance to cisplatin of A549 cells exposed to Exo/αPD1. A549 lung cancer cells were then exposed to Exo/αPD1 and Exo prior to the addition of cisplatin (CIS, 5 μM) and/or ABT263 (15 μM). ABT263 abrogated the Exo/αPD1-induced resistance to CIS (Fig. 5A).

ABT263 efficacy was then assessed in A549-inoculated xenograft mice (Fig. 5B). As expected, the CIS treatment decreased the volume of A549-induced tumors (Fig. 5C and Supplementary Fig. S5). The administration of Exo did not alter the effects of CIS on the A549 tumor, in contrast to the Exo/αPD1 inoculation which abrogated the antitumor activity of CIS. We also noted that the Exo/αPD1 inoculation decreased the Bim expression (at protein and mRNA levels) in tumors. ABT263 induced similar activity and suppressed the deleterious effect of Exo/αPD1 on the CIS-resistance of cancer cells (Fig. 5C). Our results showed that ABT263 could be used to abrogate the Exo/αPD1-induced resistance to cisplatin treatment. In addition, we observed a significant inverse correlation between the impact of treatment on tumor and the serum cytochrome c level (*r* = −0.9566; *p* = 0.0028) (Fig. 5D).

## Discussion

Recent progress in the understanding of the molecular mechanisms that govern the phenomenon of anti-PD1 resistance have made it possible to identify several major causes of this phenomenon^14,15^: evolution in the neoantigen landscape^16^, the presence of JAK1/2 mutations^17^, the presence of β-2-microglobulin mutations^18^, and the limited acquisition of memory potential on CD8 + Tcells^19,20^. While the most recent articles have identified signatures associated with or explaining the molecular mechanisms that govern the phenomenon of anti-PD1 resistance, Bertrand et al. demonstrate that the TNFα blockade overcomes resistance to anti-PD1 in a mouse experimental melanoma model and suggested that using anti-PD1 and anti-TNF antibodies could be a therapeutic solution for limiting the process of anti-PD1 resistance^21^. By identifying the T cell-derived ExomiR-4315^expression^/Bim^down-regulation^ axis as a “pan-cancer cascade” of events associated with the phenomenon of resistance to anti-PD1 therapy, our study completes the list of molecular mechanisms that govern this phenomenon. Our study is thus the first to incriminate a horizontal RNA transfer process in the transduction of the phenomenon of resistance to anti-PD1 therapy. In this horizontal RNA transfer^22^, donor cells are the T cells exposed to anti-PD1 therapy, recipient T cells are tumor cells, the exosome is the vehicle of transfer, and miR-4315 is the transferred biological information.

Over the past decade, miR research in the cancer field has identified miR as biomarkers for diagnosis, prognosis and prediction of drug efficacy, as therapeutic agents, such as onco- or tumor suppressor miR^23,24^. In this pleotropic literature, the one concerning miR-4315 appears poor. Nevertheless, miR-4315 has been identified as being upregulated in cancerous tissue compared with noncancerous breast tissue^25^, deregulated in colorectal cancer^26^ and upregulated in primary lung adenocarcinoma tissue compared with non-cancerous tissue^27^. Our work is thus the first to associate exomiR-4315 expression with a putative biomarker value for predicting the anti-cancer therapy efficacy of miR-4315.

The literature has already reported that Bim expression levels can be used to predict the response to anti-PD1 therapy in patients with metastatic melanoma^28,29^. In these articles, it is mentioned that the measurement of Bim levels in CD8^+^ T cells represents a promossing low invasive strategy to predict the response to anti-PD-1 therapy. Our study strongly differs from these findings as (1) our data involve T cells and not only CD8^+^ T cells, (2) Bim regulation occurs in tumor cells and not in immune cells, and (3) our study involves intercellular communication between T cells and cancer cells and not only T cells. In addition, our study identifies exosomal miRNA-4315 as the molecular cause of the regulation of Bim expression, while no molecular cause was underline by Dronca et al.^29^. Thus, our data are not redundant of the ones existing in the literature since they are focused on Bim in tumor cells and not on Bim in T cells.

Our work focuses on the use of 1 μg/ml of anti-PD1 antibody. The choice of this concentration was dictated by the fact that this concentration represents the minimal concentration of anti-PD1 for abolishing the detection of PD1 on the T cells (Supplementary Fig. S6). In other words, our study was carried out with the saturating concentration of anti-PD1 antibody. However, two studies reveal that the median PD-1 occupancy by anti-PD1 antibody was 64–70% depending on dose level, and was time-dependent with a mean peak occupancy of 85% (range: 70–97%) and a mean plateau occupancy of 72% (range: 59–81%) observed at 4–24 h and 57 days, respectively, after one infusion^6–30^. Based on these data, we analyzed exomiR-4315 expression following T cell exposure with anti-PD1 antibody promoting different percentages of PD1 occupancy (Supplementary Fig. S7). ExomiR-4315 expression increased with PD1 occupancy by the antibody (Supplementary Fig. S7). In this context, the equilibrium state between PD1 and the anti-PD1 antibody was enough to promote an immune response without inducing the exomiR-4315-mediated chemotherapy resistance.

Taken into consideration the fact that the PD-1 and PD-L1 inhibitors have an objective response rate of around 20–40% in a variety of metastatic tumor types, several clinical trials have investigated the effect of combined therapy including anti-PD-1 therapy and chemotherapy. If certain combinations were a success (such as the one between pembrolizumab and mFOLFOX6 for patients with advanced colorectal cancer^31^), other were failures or semi-failures. Indeed, the reading of the report made by Gadgeel et al. (the combination of pembrolizumab with carboplatin + paclitaxel or carboplatin + paclitaxel + bevacizumab or carboplatin + pemetrexed has an overall response rate of 52%, 48%, and 71%, respectively^32^) can be interpreted as a success or a semi-failure in 48 and 52% of cases fro the two first combinations. Laboratory investigations also report that combinations of chemotherapy and anti-PD-1 blockade may limit chemoresistance and progression to metastatic disease. Thus, Black et al. report that the PD-1 exposure increased ERK and mTOR phosphorylation and tumor cell proliferation^33^. Other articles report the idea that chemotherapeutic agents (temozolomide^34^, cyclophosphamide^35^, doxorubicin^36^, and cisplatin^37,38^) directly affects the immune system and alters the antitumor response. Taken into consideration all these reports, it appears that, the combinations between anti-PD-1 and chemotherapeutic agents attractive and promising, but that these combinations remain ineffective in certain patients. Our article opens the possibility to detect (by monitoring the exomiR-4315 level) certain non-responding patients to the combination of anti-PD-1 and chemotherapy.

Interestingly, ABT263 could abrogate in vitro and in a preclinical model the resistance to anti-PD1 therapy and open up new therapeutic options for bypassing this drug resistance. The “rational” nature of our proposal is based on the use of an anticancer agent frequently used in clinical trials (29 studies found for ABT263 on the ClinicalTrials.gov website) and on the detection of two biomarkers, blood exosomal-miR-4315 and cytochrome-c levels, which act as “green and red lights” for the timing of administration and the stopping ABT263 during the anti-PD1 therapy taken by the patients. In addition, due to the observation of dynamic expressions of blood exosomal-miR-4315 and cytochrome-c levels in patients treated with anti-PD1 therapy, we suggest that the use of ABT263 could be discontinuously administrated depending on the detection of biomarkers.

By using cellular and in vivo models, as well as longitudinal patient blood samples, the present work is the first to report: (i) resistance to anti-PD1 therapy involving intercellular communications mediated by exosomal miRNA; (ii) a conserved process regardless of the origin of the cancer cells receiving the T cell-derived exomiR; (iii) a resistance mechanism that could be bypassed by the administration of BH3-mimetic drugs already present in the anticancer arsenal. The use of blood epigenetic biomarkers could lead to better control of anti-PD1 therapies by making possible personalized and real time adaptation of BH3-mimetic therapy.

## Methods

### Cell culture

T cells were obtained from Stem Cell Technology (France) and were cultured in RPMI medium supplemented with 10% of fetal bovine serum, 1% penicillin–streptomycin. A172 and MCF7 cells were cultured in DMEM (4.5 g/l glucose) medium supplemented with 10% of fetal bovine serum, 1% penicillin–streptomycin. OV90 and A549 cells were cultured in RMPI medium supplemented with 10% of fetal bovine serum, 1% penicillin–streptomycin. MCF7 cells were cultured in EMEM supplemented with 10% of fetal bovine serum, 1% penicillin–streptomycin. All cells were cultivated in a 5% CO_2_ incubator at a temperature of 37 °C. MCF and OV9 cells were kindly provided by Dr P Juin (CRCINA, Nantes, France) and Dr R Spisek (Prague University, Czech Republic). A172 (CRL-1620) and A549 (CRL-185) were purchased from ATCC.

### FOXO1 activity

TransAM^®^ FKHR/FOXO1 kit (Active Motif#46396, France) was used to estimate the FOXO1 activity. Briefly, at indicated time and condition, cells were harvested and used for a protein nuclear extraction using the Nuclear Extraction Kit (Active Motif#40410, France). For each point (technical duplicate and independent biologic triplicate), 15 µg of nuclear extract were used following the Active Motif’s instructions. ELIZA plate O.D. was read on a Victor™x3 spectrophotometer (Perkin-Elmer, France).

### Cell cytotoxicity assay

Colorimetric Cell Cytotoxicity Assay Kit (Abcam#Ab112118, France) was used to estimate the cell viability. Briefly, cells were seeded in 96-well plate. After the realization of manufacturer’s instruction, absorbance was read at 570 and 605 nm using Victor™x3 spectrophotometer (Perkin-Elmer, France).

### Measurement of caspase-3 and poly ADP ribose polymerase (PARP) cleavage

Levels of cleaved caspase-3 and cleaved PARP are considered as both biomarkers of apoptosis. The measurement of these two parameters was performed using the Human Cleaved PARP1 and Human Cleaved Caspase-3 ELIZA Kits (Abcam#Ab174441 and Ab220655, France) according to the manufacturer’s protocol.

### In-cell ELIZA

In cell ELIZA were performed using the Bim Colorimetric Cell-Based ELIZA kit (Aviva Systems Biology#OKAG00554, France) according to the manufacturer’s instructions. Briefly, 5000 cells were seeded in 96-well plate to be exposed or not to indicated exosome or miRNA. After that, cells were treated with a fixing solution (4% of paraformaldehyde solution) for 10 min at room temperature. Primary antibody was incubated overnight at 4 °C. Adequate HRP-conjugated secondary antibodies were incubated for 1 h at room temperature. Detection was performed at 450 nm.

After washes, cells in each well were incubated with crystal violet solution for 5 min at room temperature, according to the manufacturer’s instructions. Absorbance was read at 595 nm, and used to normalize the “Bim signal”.

### Luciferase promoter and 3′UTR Reporter assay

Cells were seeded in 24-well plates and were transfected with the indicated firefly luciferase constructs together with an SV40-renilla control vector. Lysates were prepared at 40 h, and luciferase activity was measured using the Dual Luciferase Reporter Assay system (Promega#E1910, France) and a luminometer (MicroLumat Plus, EG&G Berthold, France).

### Cell-derived exosome isolation

After anti-PD1 exposure, T cell-derived exosomes were isolated using the ExoQuick kit (Ozyme#EXOTC10A1 or #EXOCG50A1, France) according to the manufacturer’s instructions. In brief, cell culture supernatants were harvested and centrifuged at 3000 *g*/15 min. The ExoQuick solution was incubated with supernatant at 4 °C/overnight. After a first centrifugation (1500 *g*/30 min), the supernatant was aspirated and the residual solution was centrifuged (1500 *g*/5 min). The exosome pellet was resuspended in PBS. We measured purified exosome total protein concentrations using the Bradford assay (Bio-Rad Laboratories, France), and purified exosomes were stored at −80 °C until use.

Nanosight experiments indicated that Exoquick preparation is mainly composed of extracellular vescules included in a size range of 80–120 nm i.e., a size range defining exosome (Additional file 1). Consequently, the term “exosome” was used in this article.

### Cell treatment with exosomes and/or miR

At T0, 7 × 10^5^ cells were seeded in a 12-well plate. After 1 day, cells were co-incubated with T cell-derived exosomes (150 μg) and α-amanitin (50 μg/ml) (Sigma#A2263, France). The residual exosomes were eliminated via three cell washes in PBS solution. α-amanitin was used to block the putative miRNA transcription caused by experimental conditions since α-amanitin blocks the DNA-dependent RNA polymerase II activity^39^. The exosome-delivered quantity of miRNA in the cells was estimated using qRT-PCR by means of the difference in Ct value between α-amanitin-treated cells with or without exosomes.

### Exosome loading and cell transfection with anti-miRNA

The exosomes were transfected with anti-miRNA using Exo-Fect Exosome Transfection reagent (Ozyme#EXFT20A-1, France). Briefly, anti-miRNA was incubated with the exosomes (300 μg of exosomal protein) in a shaker for 15 min at 37 °C. After the addition of ExoQuick-TC solution to stop the reaction, the mixture was incubated on ice for 30 min. After centrifugation, the transfected exosome pellet was resuspended in 300 μl of PBS before use. We measured purified exosome total protein concentrations using the Bradford assay (Bio-Rad Laboratories, France).

HiPerFect Transfection Reagents (Qiagen#301705, France) were used for the transfection of cells with mimic-miR, mimic-mutated-miR and miR inhibitor (also named anti-miR). These reagents are mimic-miR-4315: 5′CCGCUUUCUGAGCUGGAC (Syn-hsa-miR-4315 miScript miRNA Mimic), mimic-mutated-miR-4315: 5′CCGAAAUCUGAGCUGGAC, and anti-miR-4315 (miScript miRNA Inhibitor and miScript Inhibitor Neg. Control (Qiagen, France).

### Plasma samples

Plasma was collected from GBM patients treated at the “Institut de Cancérologie de l’Ouest” (ICO, http://www.ico-cancer.fr). All patients recruited gave signed, informed consent. All the samples collected and the associated clinical information were registered in the database (N° DC-2018-3321) validated by the French research ministry. Biological resources were stored at the “Centre de Ressources Biologiques-Tumorothèque” (Institut de Cancérologie de l’Ouest, Saint-Herblain, F44800, France).

### Isolating exosomal miRNA from blood

From the blood sample collected in K + EDTA tubes, 4–5 ml of plasma was isolated via two centrifugations (10 min/1900 *g*/4 °C and 10 min/16,000 *g*/4 °C) of 10 ml whole blood. 1 ml of plasma was processed for the isolation of miRNA using the ExomiRNeasy serum/plasma kit (Qiagen#217184, France) according to the manufacturers instructions.

### miRNA RT-qPCR

miScript II RT with miScriptHiSpec buffer (#218161), miScript SYBR Green PCR kits (#2180300), and miScript Primer Assays (#218300) Qiagen, France) were used to perform the RT-qPCR on the Rotor-Gene Q (Qiagen, France). Quantification and the purity of the miRNA were analyzed using Qubit (Thermo, France) and Agilent 2100 (Small RNA kit, Agilent#5067-1548, France) respectively, according to the manufacturer’s instructions, respectively.

### RT-qPCR analysis

RNA extract is performed using RNeasy Mini QIAcube Kit and QIAcube (Qiagen#1038703, France). RT-qPCRs are performed using QuantiTect Reverse Transcription Kit (#205313), QuantiFast SYBR Green PCR Kit (#204057), QuantiTect Primer Assays (#249900) and Rotor-Gene Q as real-time thermocycler (Qiagen, France). Reference gene RPLP0 was used, with the 2^−∆∆Ct^ relative quantification method.

### In vivo experiments

The experimental procedures using animals were in accordance with Institutional Animal Care guidelines and the French National Committee of Ethics. In addition, all experiments were conducted according to the Regulations for Animal Experimentation at the “Plate-forme Animalerie” in the “Institut de Recherche en Santé de l’Université de Nantes (IRS-UN)” and approved by the French National Committee of Ethics. Cultured A549 cells were harvested by trypsinization, washed and resuspended in saline buffer. Cell suspensions were injected subcutaneously (s.c.) into the flanks of 7-/8-week-old MNRI Nude mice (Janvier Labs, France). Tumor volume based on caliper measurements was calculated using the modified ellipsoidal formula (Tumor volume = 1/2(length × width^2^)).

### Statistical analysis and results

Except when indicated, data are representative of the mean and standard deviation calculated from three independent experiments. Significance of the differences in means ± standard deviations was calculated using the Student-t test. The significance of correlation between two parameters was calculated using Pearson’s test. *p* < 0.05 was used as a criterion for statistical significance.

## Supplementary information

Legends of supplementary figures

Supplementary figure 1

Supplementary figure 2

Supplementary figure 3

Supplementary figure 4

Supplementary figure 5

Supplementary figure 6

Supplementary figure 7
