# Supplementary material for: Anti-PD1 therapy induces lymphocyte-derived exosomal miRNA-4315 release inhibiting Bim-mediated apoptosis of tumor cells
Source: Cell Death Dis. 2020 Dec 11;11(12):1048. doi: 10.1038/s41419-020-03224-z (PMC7733505; doi:10.1038/s41419-020-03224-z)
Supplement: Supplementary file 1 — Legends of supplementary figures [file 41419_2020_3224_MOESM1_ESM.docx]

**Anti-PD1 therapy induces lymphocyte-derived exosomal miRNA-4315 release inhibiting Bim-mediated apoptosis of tumor cells.**

Nina Guyon^1,2,3,4^, Delphine Garnier^1,2,3,4^, Joséphine Briand^1,2,3,4^, Arulraj Nadaradjane^1,2,3,4^, Gwenola Bougras-Cartron^1,2,3,4^, Judith Raimbourg^1,2,6^, Mario Campone^6^, Dominique Heymann^1,2^, François M Vallette^1,2,3,4,5^, Jean-Sébastien Frenel^1,2,6^ and Pierre-François Cartron^1,2,3,4,5*^

^1^ CRCINA, INSERM, Université de Nantes, Nantes, France.

^2^ Equipe Apoptose et Progression tumorale, LaBCT, Institut de Cancérologie de l'Ouest, Saint Herblain, France.

^3^ Cancéropole Grand-Ouest, réseau Epigénétique (RepiCGO).

^4^ EpiSAVMEN Consortium (Région Pays de la Loire).

^5^ LabEX IGO, Université de Nantes, France.

^6^ Department of Medical Oncology, Institut de cancérologie de l'Ouest site René Gauducheau, Saint Herblain, France.

^*^Correspondence: [pierre-francois.cartron@inserm.fr](mailto:pierre-francois.cartron@inserm.fr), CRCINA, INSERM U1232, Equipe Apoptose et Progression tumorale, LaBCT, Institut de Cancérologie de l'Ouest, Boulevard du Pr Jacques Monod, Saint Herblain, 44805, France.

**Running title:** Exosomal miRNA-4315 as a signature of resistance to anti-PD1 therapy

**Keywords:** Epigenetic, exosomal miRNA, anti-PD1 therapy, Bim, BH3 mimetic, acquired resistance.

**Figure S1. Schematic representation of predicted binding sequence between Bim 3’UTR sequence and miR-4315 (wild type) or the mutated form of miR-4315.**

Predicted binding sequence are in grey in Bim 3’UTR sequence and in blue in wild type and mutated miR-4315.

**Figure S2. Impact of exosomal pretreatment on the cell death induction by oxaliplatin or paclitaxel on ovarian (OV90) and breast (MCF7) cancer cells.**

**Figure S3. Schematic representation of blood tests performed in patients.**

**Figure S4. Correlation between the exomiR-4315 and serum cytochrom c levels.**

**Figure S5. Graphs report the Bim expression levels, serum cytochrome c levels and the tumor volumes for each mice considerated in our study**.

In « tumor volume » graph, histograms in light color represent the volume of established tumors and histograms in dark color represent the tumor volume at the end of treatments.

**Figure S6. Curve illustrates the percentage of occupency of anti-PD-1 on PD-1 receptor in function of the amount of anti-PD-1 incubated.**

**Figure S7. Relationship between the level of ExomiR-4315 expression and the percentage of anti-PD-1 occupancy on PD-1 receptor**
