# Supplementary figures and images for: Anti-PD1 therapy induces lymphocyte-derived exosomal miRNA-4315 release inhibiting Bim-mediated apoptosis of tumor cells
Source: Cell Death Dis. 2020 Dec 11;11(12):1048. doi: 10.1038/s41419-020-03224-z (PMC7733505; doi:10.1038/s41419-020-03224-z)

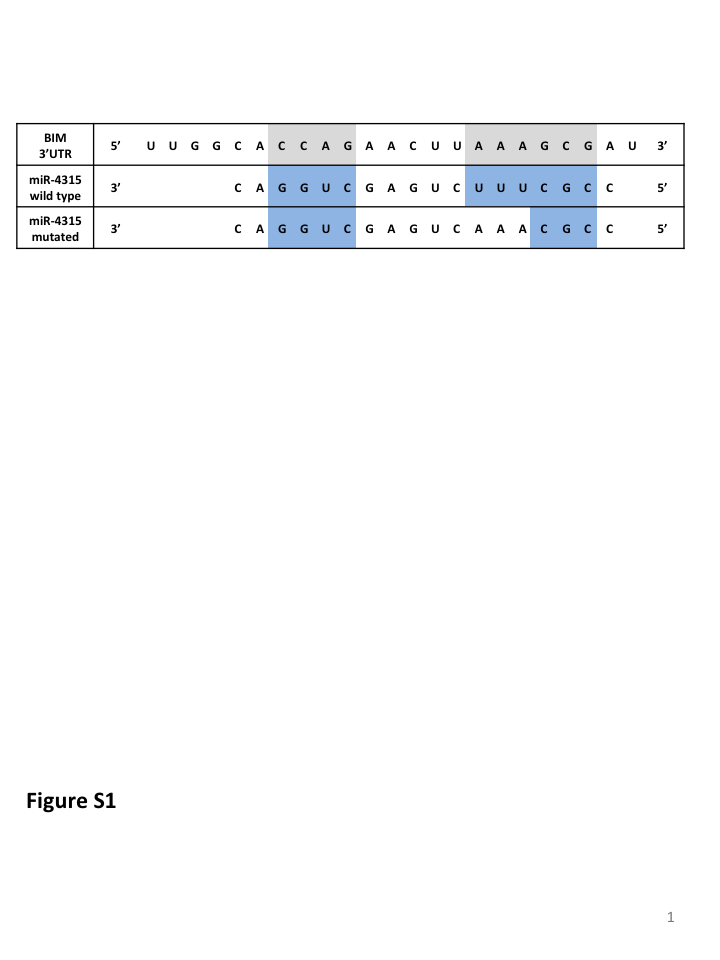

Supplement: Supplementary file 2 — Supplementary figure 1 [file 41419_2020_3224_MOESM2_ESM.png]

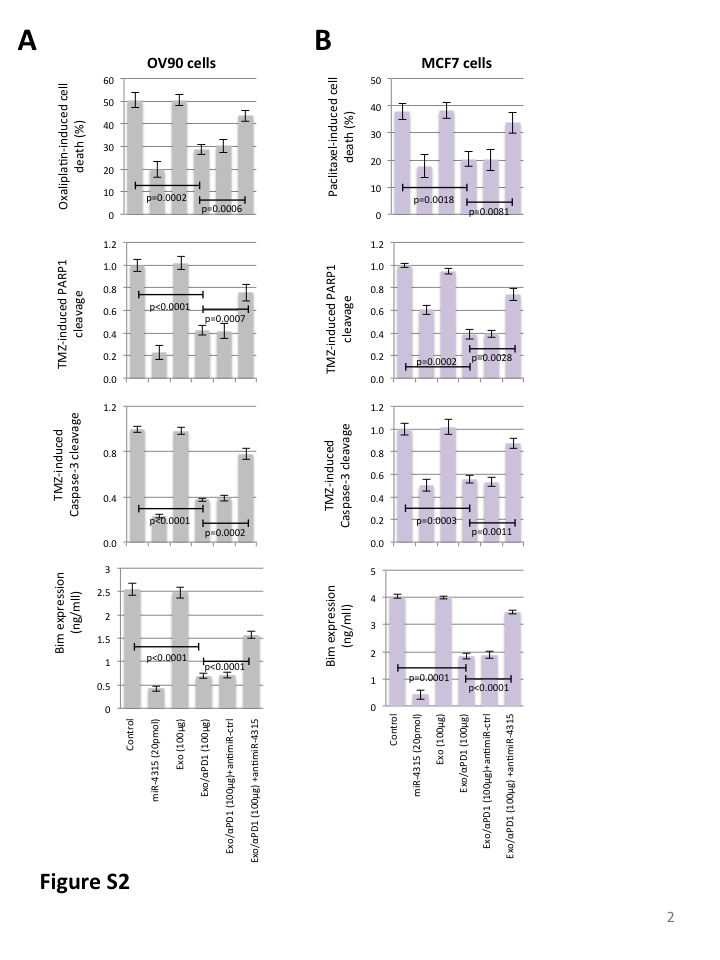

Supplement: Supplementary file 3 — Supplementary figure 2 [file 41419_2020_3224_MOESM3_ESM.png]

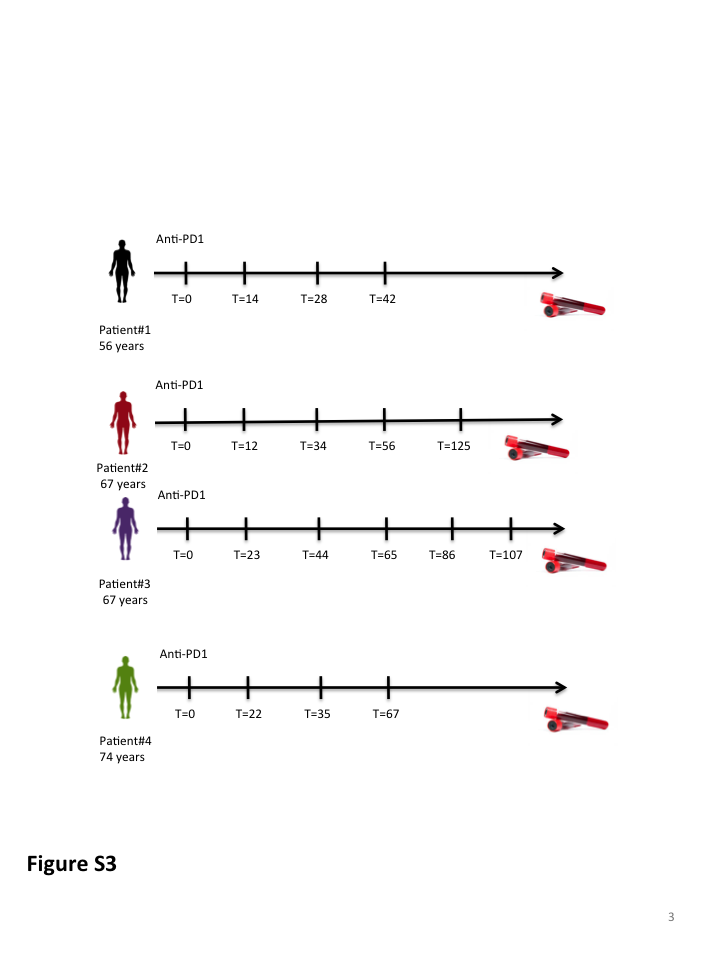

Supplement: Supplementary file 4 — Supplementary figure 3 [file 41419_2020_3224_MOESM4_ESM.png]

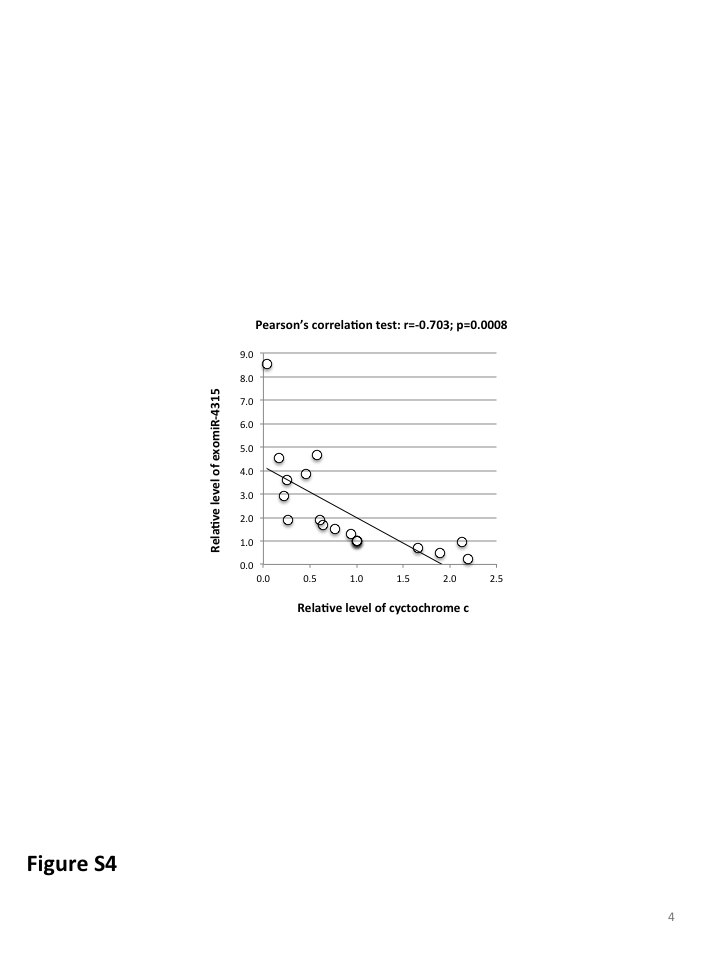

Supplement: Supplementary file 5 — Supplementary figure 4 [file 41419_2020_3224_MOESM5_ESM.png]

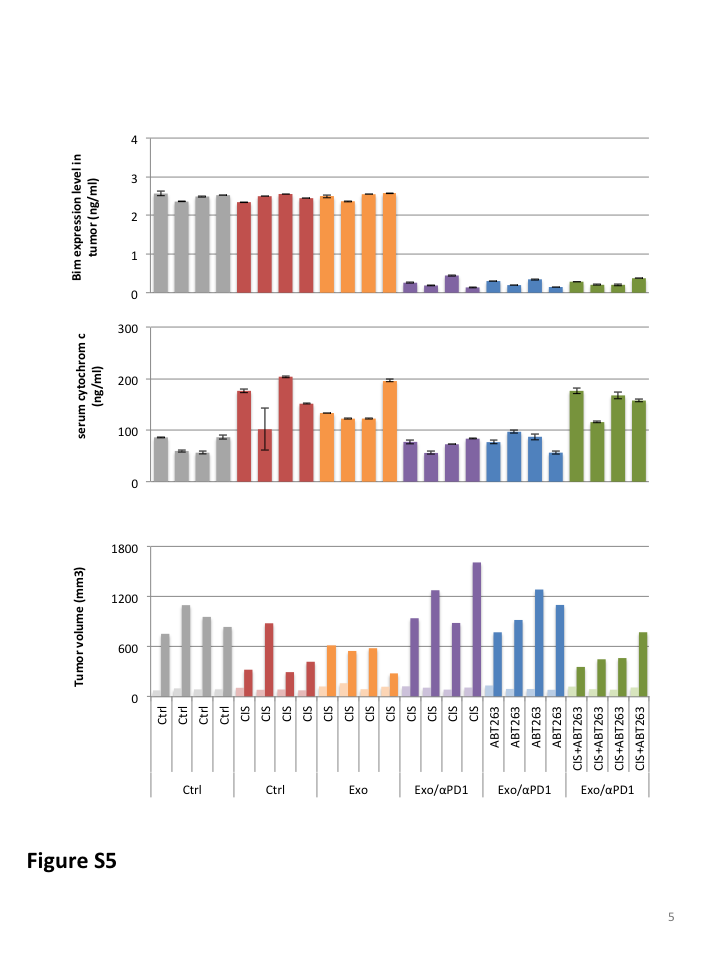

Supplement: Supplementary file 6 — Supplementary figure 5 [file 41419_2020_3224_MOESM6_ESM.png]

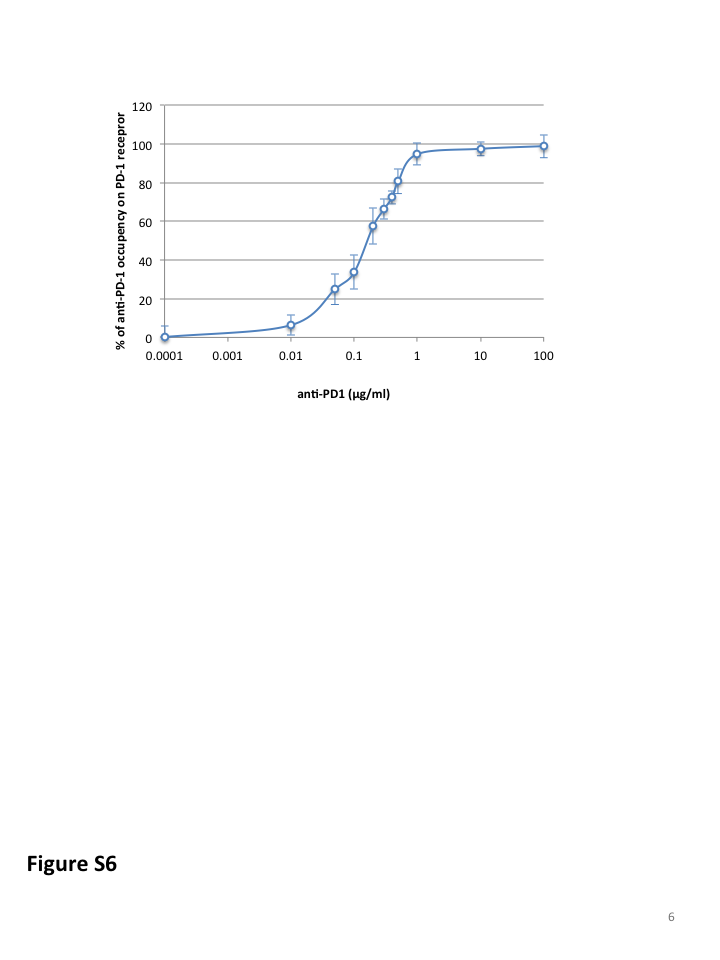

Supplement: Supplementary file 7 — Supplementary figure 6 [file 41419_2020_3224_MOESM7_ESM.png]

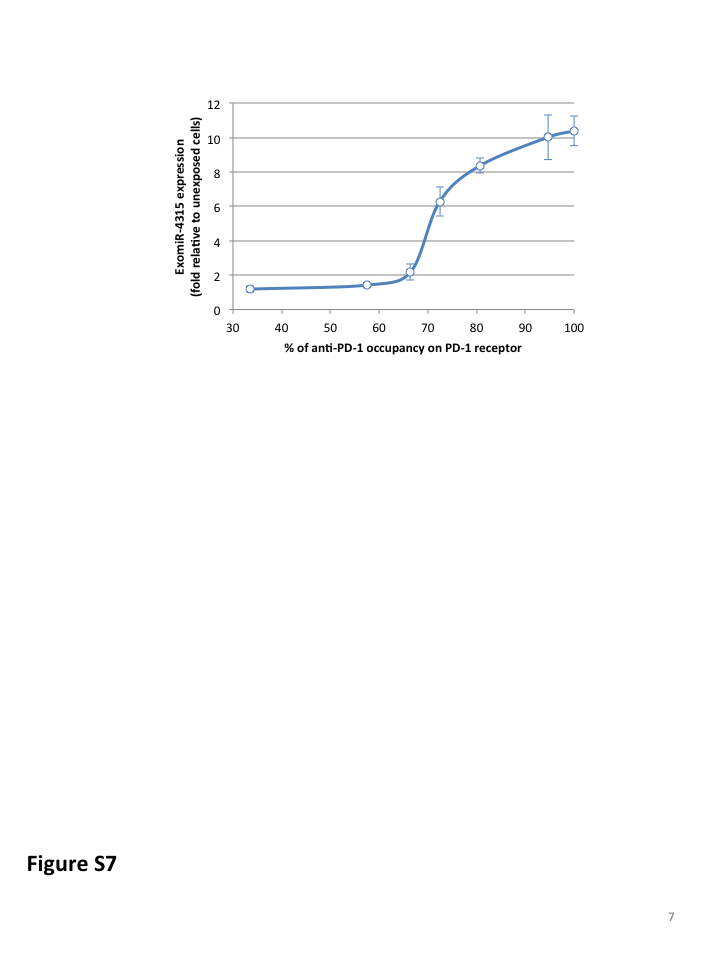

Supplement: Supplementary file 8 — Supplementary figure 7 [file 41419_2020_3224_MOESM8_ESM.png]
